# Development of sustainable gel systems with thermo–sensitive properties by enzymatic modification of upcycled rice cake flour

**DOI:** 10.1016/j.fochx.2026.104095

**Published:** 2026-06-12

**Authors:** Da-Hee Kim, Tae-Ho Choi, Ji-Hoon Park, Su-Hyeon Kim, Seo-Jin Jeon, Hyun-Mo Jeong, Jae-Hoon Shim

**Affiliations:** a Department of Food Science and Nutrition, and the Korean Institute of Nutrition, Hallym University, Hallymdaehak-gil 1, Chuncheon-si, Gangwon-do 24252, Republic of Korea; b Department of Food Science and Nutrition, Kangwon National University, Gangneung Campus, 7 Jukheon-gil, Gangneung-si, Gangwon-do 25457, Republic of Korea

**Keywords:** Cyclodextrin glucanotransferase, Freeze–thaw stability, Modified starch, Rice cake, Thermo–sensitive gel, Upcycling

## Abstract

Rice cake flour (RCF), obtained by milling byproducts generated during rice cake production, was enzymatically modified using cyclodextrin glucanotransferase (CGTase) to develop thermo–sensitive gels (TSGs). Samples prepared at different CGTase dosages were designated as TSG 40, TSG 60, TSG 80, and TSG 100. CGTase treatment redistributed starch branch–chain lengths, reducing short chains (DP 6–15) while increasing medium chains (DP 16–30), and decreased the weight–average molecular weight from 8.78 × 10^7^ Da to 2.48–4.86 × 10^7^ Da. These structural changes likely contributed to reversible viscoelastic behavior during repeated heating–cooling cycles through modification of starch chain organization. TSG 80 exhibited storage modulus (G′) recovery exceeding 109%, while TSG 60 showed improved freeze–thaw stability with reduced syneresis (4.65%) compared to native RCF (9.35%). Texture profile analysis showed reduced hardness with no change in cohesiveness. CGTase-mediated chain rearrangement improved rheological behavior and freeze–thaw stability of upcycled starch gels, highlighting their potential for thermo-processed and frozen food systems.

## Highlights

• CGTase treatment valorized surplus rice cake flour into thermo-sensitive gels (TSG).
• Enzymatic modification reduced Mw and redistributed starch branch-chain lengths
• Structural modification enabled reversible viscoelastic behavior in TSGs.
• Moderate enzyme treatment improved freeze–thaw stability in TSGs.
• HPAEC, HPSEC, TPA, and SEM revealed structural factors affecting gel stability.

## 1 Introduction

Food processing by–products represent a significant challenge in terms of resource inefficiency and environmental impact (Mateos-Aparicio & Matias, 2019). With increasing demand for processed foods, substantial amounts of edible materials are discarded due to overproduction or failure to meet quality standards, highlighting the need for effective upcycling strategies (Peira et al., 2018; Torres-León et al., 2018).

In Korea and across Asian food industries, rice cake production has expanded rapidly in both domestic and international markets, driven by the growing popularity of products such as tteokbokki (Han & Kwon, 2023; Jin et al., 2024; Moon & Cha, 2023). As production increases, non–saleable rice cake fragments are inevitably generated during processing steps such as cutting and molding. Although these fragments are nutritionally equivalent to the final products, they are excluded from commercial distribution due to irregular size or appearance and are typically discarded. Upcycling these by–products into value–added materials offers a promising strategy for improving resource efficiency (Wibisono et al., 2025). Notably, these fragments are produced under standardized manufacturing conditions, resulting in consistent composition and processing history, which enhances their suitability for structured upcycling applications (H.-S. Lee & Jang, 2008).

In this study, non–marketable rice cake fragments were utilized as a base material and converted into surplus rice cake flour (RCF) through a controlled drying and milling process. Rice cake is produced from rice flour and water, with starch as the major constituent. In general, rice flour contains approximately 80–90% starch on a dry basis (Rhowell & Nese, 2021), indicating that the functional properties of RCF are largely governed by starch structure and organization. During rice cake manufacturing, starch undergoes partial gelatinization followed by retrogradation, which alters its crystalline organization and reduces the reversibility of gel formation as well as freeze–thaw stability (Eom et al., 2018; Wang et al., 2024). Therefore, developing processing or modification strategies that can improve the structural stability and functional performance of upcycled RCF is essential for enabling its effective utilization in food and polysaccharide–based material applications. However, its poor solubility and unstable physicochemical properties still pose challenges for high–value utilization, necessitating effective modification strategies (Gong et al., 2024).

Starch is the major component of rice cake flour, and its structural organization plays a critical role in determining physicochemical properties and gelation behavior (Seo et al., 2007; Varghese et al., 2022). Various modification strategies, including physical, chemical, and enzymatic approaches, have been explored to improve these properties (Gong et al., 2024). Among these, enzymatic modification offers distinct advantages, such as mild reaction conditions, environmental compatibility, and the ability to selectively alter starch structure (Miao & BeMiller, 2023). Notably, enzymatic treatment can induce thermo–responsive behavior, enabling the formation of gels that exhibit reversible viscoelastic transitions in response to temperature changes (Xiao et al., 2025). However, studies on utilizing food–processing by–products such as rice cake flour for the development of thermo–sensitive starch gels remain limited.

Cyclodextrin glucanotransferase (CGTase; EC 2.4.1.19), a member of the α–amylase superfamily, catalyzes various transglycosylation reactions, including disproportionation, cyclization, and coupling, as well as hydrolysis, thereby modifying glucan chains in starch (Leemhuis et al., 2010). These reactions induce redistribution of chain lengths and partial cleavage of starch molecules, leading to the formation of cluster-like structures, which in turn alter molecular weight distribution and consequently affect physicochemical properties such as solubility, flexibility, and structural stability (Kim et al., 2023). Among these, the disproportionation reaction plays a key role by modifying chain length distribution and molecular architecture, which directly influences gel viscoelasticity and thermal behavior (Kobayashi et al., 1999). Due to its reaction specificity and established safety, CGTase is widely used as a food–grade enzyme for starch modification and the production of functional carbohydrates (Pardhi et al., 2023). This multifunctional catalytic capability makes CGTase particularly suitable for tailoring the rheological and thermal properties of starch–based gel systems.

Thermo–sensitive gels (TSGs) are materials that exhibit reversible changes in viscoelastic properties in response to temperature variations (Fan et al., 2022). Such stimulus–responsive behavior enables dynamic modulation of gel structure and mechanical properties, making TSGs attractive for food and polysaccharide–based material applications (Klouda & Mikos, 2008; Zhao et al., 2024). In particular, starch–based systems have gained attention as sustainable alternatives to conventional gels due to their biocompatibility and non–toxicity. Enzymatic approaches offer additional advantages, including mild processing conditions and compatibility with industrial applications (Karim & Bhat, 2008). Although thermo–sensitive starch gels have been explored using physical, chemical, and enzymatic modifications, studies on CGTase–based systems remain limited. In particular, the role of CGTase–mediated disproportionation in inducing thermo–responsive behavior through controlled chain rearrangement has not been fully elucidated. Therefore, applying CGTase to develop TSGs from upcycled starch sources represents a novel and practical strategy for developing thermo–sensitive starch–based gels from upcycled materials.

In this study, RCF was enzymatically modified using CGTase to induce rearrangement of starch molecular chains and to develop TSGs with enhanced structural stability and temperature–dependent rheological behavior. This study aims to establish an effective modification strategy for improving the functional properties of upcycled RCF and to evaluate its potential applicability in thermo–processed and frozen food systems, as well as in sustainable polysaccharide–based materials such as biofilms and gel matrices (Lv et al., 2025).

## 2 Materials and methods

### 2.1 Materials and reagents

RCF was provided by SEJUN F&B (Hongcheon, Korea). Cyclodextrin glucanotransferase (CGTase; Toruzyme Neo) was purchased from Novozymes (Bagsvaerd, Denmark). Isoamylase was obtained from Megazyme (Wicklow, Ireland). Glucose (G1), maltose (G2), maltotriose (G3), and sodium acetate were supplied by Sigma–Aldrich (St. Louis, MO, USA). Maltotetraose (G4) was purchased from Tokyo Chemical Industry Co., Ltd. (Tokyo, Japan). Maltopentaose (G5), maltohexaose (G6), and maltoheptaose (G7) were obtained from CarboExpert Inc. (Daejeon, Korea). Certified sodium hydroxide solution was purchased from Thermo Fisher Chemical (Waltham, MA, USA).

### 2.2 Preparation of TSGs

To prepare TSGs, a 10% (*w*/*v*) suspension of rice cake flour (RCF) was gelatinized by heating at boiling temperature (∼100 °C) under continuous stirring in a reactor. The gelatinized slurry was cooled to 60 °C, and CGTase was added at concentrations of 0.4 × 10^−4^, 0.6 × 10^−4^, 0.8 × 10^−4^, and 1.0 × 10^−4^ U/g RCF. The reaction mixture was maintained at 60 °C with stirring for 20 min to allow enzymatic modification, after which the enzyme was inactivated by heating at boiling temperature. The samples were then gelled at 4 °C for at least 12 h, followed by freezing at −80 °C for 24 h and lyophilization using a benchtop freeze–dryer (FDB–5503, OPERON, Gimpo, South Korea). Samples prepared under these conditions were designated as TSG 40, TSG 60, TSG 80, and TSG 100, respectively.

### 2.3 Thermo–sensitive rheological behavior of TSGs

The rheological properties of TSGs were measured using a rheometer (Discovery HR 10, TA Instruments, New Castle, DE, USA) equipped with a parallel–plate system (40 mm diameter) and a 1000 μm gap. Gel samples were placed on the lower plate, compressed to the specified gap with the upper plate, and the excess material was carefully trimmed. To prevent dehydration during measurement, a thin layer of silicone oil was applied around the sample edges. Dynamic rheological properties, including storage modulus (G′) and loss modulus (G″), were recorded during heating and cooling cycles ranging from 4 °C to 100 °C. Measurements were conducted at a constant frequency of 1 Hz and strain of 0.5%, with the temperature varied at a rate of 3 °C/min. Each sample was subjected to three consecutive temperature cycles, consisting of heating to 100 °C, held for 2 min, followed by cooling to 4 °C and held for 30 min. All measurements were performed within the linear viscoelastic region (LVR).

### 2.4 High–performance anion exchange chromatography (HPAEC) analysis

The side–chain–length distribution of TSGs was analyzed by high–performance anion–exchange chromatography (HPAEC) using a previously established protocol (H.-W. Lee et al., 2016). Starch samples (1%, *w*/*v*) were gelatinized, mixed with two volumes of ethanol, and precipitated at −20 °C for 3 h. The mixture was centrifuged at 11,000 ×*g* for 15 min at 4 °C, and the supernatant was discarded; this step was repeated 3 time. The remaining pellet was dried using a vacuum concentrator (ScanVac, LaboGene, Lillerød, Denmark) and a dry oven to remove residual solvent. The dried pellet was resuspended in 100 mM sodium acetate buffer (pH 4.5), heated to 100 °C, and cooled to room temperature. Isoamylase (20 U/mg substrate) was added, and the sample was incubated at 40 °C for 96 h for complete debranching. The reaction was terminated by heating in a boiling water bath for 10 min. HPAEC was performed on a Dionex system (Sunnyvale, CA, USA) equipped with a guard column (CarboPac PA1, 4 × 50 mm), an analytical column (CarboPac PA1, 4 × 250 mm), and a pulsed amperometric detector (PAD; ED40, Dionex, Sunnyvale, CA, USA). An injection volume of 20 μL was used, and separation was achieved at a flow rate of 1.0 mL/min using an elution gradient of sodium acetate in 150 mM NaOH.

### 2.5 High–performance size exclusion chromatography (HPSEC) analysis

Molecular weight changes induced by enzymatic treatment were analyzed by HPSEC. A starch suspension was prepared in distilled water at 2.5% (*w*/*v*) and hydrated for 30 min. Dimethyl sulfoxide (DMSO) was then added to obtain a final concentration of 1% (w/v). The mixture was heated at 100 °C for 1 h and stirred at room temperature overnight. Samples were mixed with ethanol (1: 6, sample: ethanol), centrifuged to remove the supernatant, and the precipitate was dispersed in a boiling water bath for 30 min. The final preparation was filtered through a 5 μm syringe filter (Acrodisc 25 mm, Pall Co., Port Washington, NY, USA) prior to injection. HPSEC was performed on an HPLC–Agilent 1100 system (Agilent Technologies, Santa Clara, CA, USA) equipped with a degasser, an autosampler, a pump (Waters 510; Waters Co., Milford, MA, USA), and SEC columns (Shodex SB–806HQ and Shodex SB–804HQ OHpak, Showa Denko, Tokyo, Japan). The column temperature was maintained at 5 °C. The flow rate was set to 0.6 mL/min, and detection was carried out with a multi–angle light scattering (MALS) detector (Dawn Heleos–II, Wyatt Technology, Santa Barbara, CA, USA) maintained at 50 °C.

### 2.6 Texture profile analysis (TPA) of TSGs

The texture parameters of TSGs were evaluated using a TPA (Texture Analyzer TX–700, Lamy Rheology, Champagne au Mont d'Or, France). Immediately after terminating the enzymatic reaction, samples were cast into cylindrical molds with a diameter of 5 cm, cooled to induce gelation, and prepared as disks with a thickness of 1.5 cm and a diameter of 5 cm. TPA measurements were conducted in a two–cycle compression mode with a tester equipped with a 500 N load cell, using a flat probe with a diameter of 50 mm. The test speed was set to 1 mm/s, the return speed to 1 mm/s, the sample detection threshold to 0.05 N, and the compression distance to 50%. Measurements were performed in triplicate for each sample to ensure statistical reliability.

### 2.7 Scanning electron microscope (SEM) analysis

Surface microstructures were examined using a SEM (PE–100, ModulSci, Daejeon, Korea). Freeze–dried TSG samples were sectioned to obtain cross sections and mounted on aluminum cylinder stub (25 mm × 10 mm) with carbon tape. A platinum (Pt) coating was applied using an ion sputter coater (G20, G SEM, Suwon, Korea) at a sputtering current of 10 mA for 90 s. SEM observations were performed at accelerating voltages of 5 and 10 kV at 500× magnification.

### 2.8 Freeze–thaw stability

Freeze–thaw stability was assessed by modifying a previously reported method (K. Lee et al., 2006). Immediately after enzymatic reaction, TSGs were transferred to 50 mL tubes and allowed to gel at 4 °C. The initial mass (S0) of each sample was recorded. Samples were stored at −80 °C for 18 h and thawed in a 25 °C water bath for 60 min. This freeze–thaw procedure was repeated for three cycles to evaluate the stability of the gels under repeated freezing and thawing conditions. Following each freeze–thaw cycle, samples were centrifuged at 3787 ×*g* for 10 min at 20 °C, the supernatant was removed, and the residual gel mass (SR) was recorded.

$$\text{Syneresis}\;\left(\%\right)=\left(S0-\mathrm{SR}\right)/S0\times 100$$

### 2.9 Statistical analysis

All experiments were performed in three independent replicates. Statistical analyses were conducted using SPSS (version 25.0; IBM Corp., Armonk, NY, USA). Statistical significance was assessed at *P* < 0.05, and differences among groups were analyzed using Duncan's multiple range test.

## 3 Results

### 3.1 Rheological behavior of RCF and TSGs under temperature cycling

**Fig. 1:**
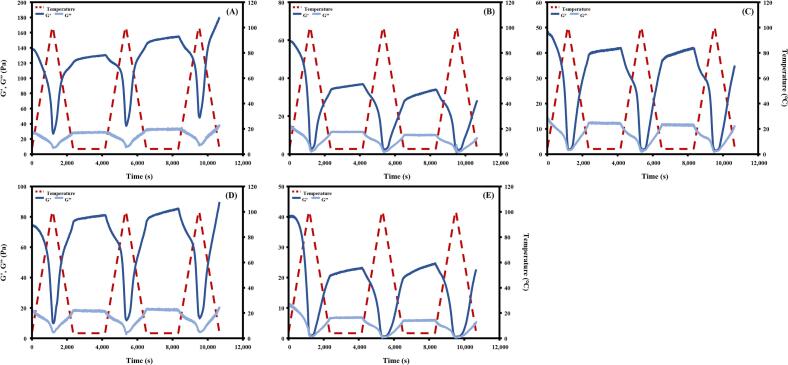
Heating and cooling cycle dependent changes in storage modulus and loss modulus. G′ and G″ were measured during temperature cycling with repeated heating and cooling. (A); untreated RCF, (B); CGTase treated TSG 40 (0.4 × 10^−4^ U/g substrate), (C); CGTase treated TSG 60 (0.6 × 10^−4^ U/g substrate), (D); CGTase treated TSG 80 (0.8 × 10^−4^ U/g substrate), (E); CGTase treated TSG 100 (1.0 × 10^−4^ U/g substrate).

To investigate rheological transitions of RCF and TSGs during temperature cycling, the storage modulus (G′) and loss modulus (G″) were monitored. Native RCF showed progressive decreases in G′ during the first, second, and third heating ramps, declining by 78.82%, 71.96%, and 63.93%, respectively, yet exhibited substantial recovery during cooling, with G′ increasing by 92.92% in the first and 116.39% in the second cooling step, surpassing the initial value in the latter (Fig. 1A).

All enzyme–treated samples (TSGs) also exhibited pronounced reductions in G′ during heating, typically exceeding 85–98% depending on enzyme level (Fig. 1B–1E). For TSG 40, G′ declined sharply during heating, decreasing by 93.93%, 95.96%, and 98.54% across the three ramps, and partially recovered during cooling, increasing by 60.08% and 55.61% in the first and second cycles (Fig. 1B). TSG 60 showed similar behavior, with G′ reductions of 95.60%, 96.78%, and 96.81%, followed by increases of 86.09% and 86.44% during cooling (Fig. 1C). TSG 80 exhibited the strongest structural recovery, with increases of 109.54% and 116.62% in the first and second cooling cycles, both exceeding the initial modulus. Across all samples, G″ showed trends similar to those of G′, indicating concurrent changes in viscous and elastic components. In TSG 40, 60, and 100, G′ and G″ converged at high temperatures, suggesting a transition toward a less structured state.

**Fig. 2:**
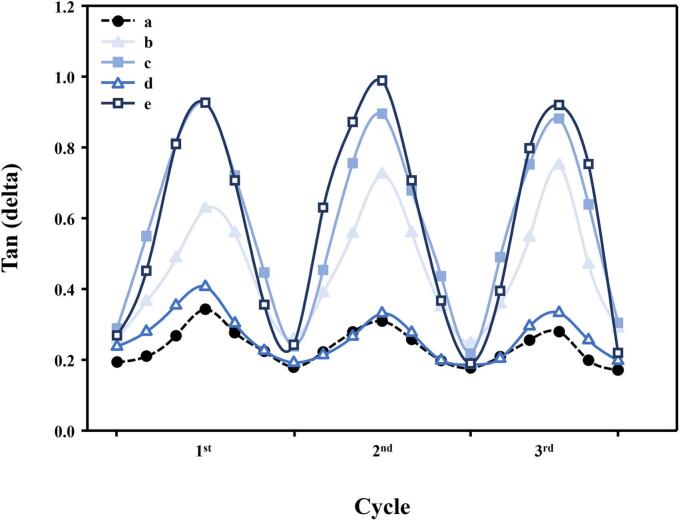
Tan δ profiles of RCF and CGTase treated TSGs during repeated heating–cooling cycles. Tan δ responses of untreated RCF (a) and TSGs prepared at enzyme levels designated as TSG 40 (b), TSG 60 (c), TSG 80 (d), and TSG 100 (e). Tan δ values were recorded at the end of each heating and cooling ramp over three consecutive thermal cycles. The maximum and minimum tan δ values correspond to the terminal points of the heating and cooling phases, respectively.

Tan delta (tan δ), defined as G″/G′, was used to evaluate the relative contributions of viscous and elastic behavior. The maximum tan δ values during heating were 0.34 for RCF, 0.75 for TSG 40, 0.93 for TSG 60, 0.40 for TSG 80, and 0.99 for TSG 100. All samples remained within the tan δ < 1 range, while enzyme–treated samples generally showed higher tan δ than RCF. This indicates a relative increase in viscous behavior during heating. In TSG 40, 60, and 100, tan δ approached 1 at the end of heating. TSG 40 and TSG 60 exhibited relatively small intra–cycle variations and consistent G′ and G″ values across repeated cycles, indicating stable viscoelastic behavior during temperature cycling (Fig. 2).

### 3.2 Side chain length distribution

**Fig. 3:**
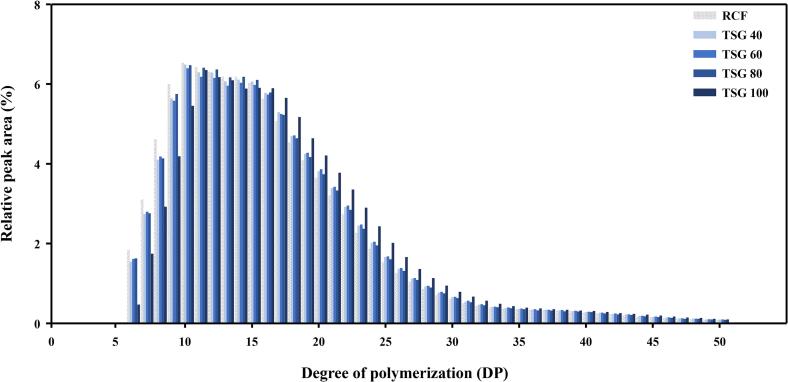
Comparative side chain length distributions of RCF and CGTase–treated TSGs. Debranched starch samples were analyzed by HPAEC, and relative peak areas were calculated across a range of degrees of polymerization (DP).

HPAEC analysis showed differences in side–chain length distribution between untreated RCF and CGTase–modified TSGs (Fig. 3). In the short–branch region (DP 6–15), all enzyme–treated samples exhibited markedly lower relative peak areas compared with RCF, indicating a reduction in short branches. In contrast, intermediate branches (DP 16–30) increased in all modified samples, with the most pronounced increase observed in TSG 100. For long branches (DP ≥ 31), most samples showed proportions similar to RCF; however, TSG 100 displayed a notably higher proportion of long chains. These changes became more pronounced with increasing enzyme level.

### 3.3 Comparison of average molecular weight

HPSEC revealed substantial differences in the weight–average molecular weight (Mw) between untreated RCF and CGTase–treated samples (Table 1). Native RCF exhibited the highest Mw (8.78 × 10^7^ Da). In contrast, all enzyme–treated samples showed markedly lower molecular weights, with TSG 40 and TSG 60 displaying Mw values of 2.48 × 10^7^ Da and 2.92 × 10^7^ Da, respectively. At higher enzyme levels, TSG 80 and TSG 100 exhibited moderate increases in Mw, reaching 4.86 × 10^7^ Da and 4.48 × 10^7^ Da, respectively. Overall, Mw decreased following CGTase treatment, with partial increases observed at higher enzyme levels.

**Table 1:** Changes in average molecular weight of RCF following CGTase treatment.

Sample	M_n_ (Da)	M_w_ (Da)	Mw/Mn
RCF	7.76 × 10^7^	8.78 × 10^7^	1.13
TSG 40	2.45 × 10^7^	2.48 × 10^7^	1.01
TSG 60	2.88 × 10^7^	2.92 × 10^7^	1.01
TSG 80	4.81 × 10^7^	4.86 × 10^7^	1.01
TSG 100	4.44 × 10^7^	4.48 × 10^7^	1.01

### 3.4 Gel texture profile of TSGs

**Fig. 4:**
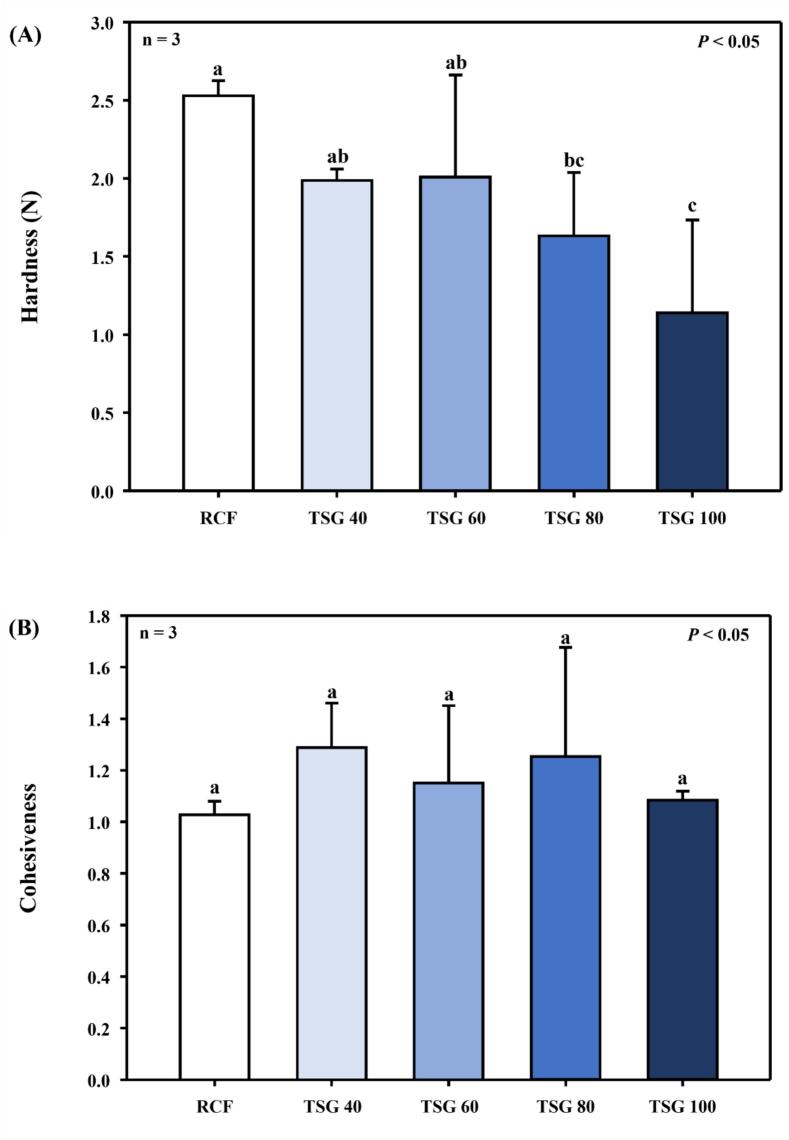
TPA of RCF and CGTase–treated TSGs. (A) Hardness and (B) cohesiveness of starch gels prepared at different CGTase levels. RCF; untreated rice cake flour, TSG 40; CGTase–treated (0.4 × 10^−4^ U/g substrate), TSG 60; CGTase–treated (0.6 × 10^−4^ U/g substrate), TSG 80; CGTase–treated (0.8 × 10^−4^ U/g substrate), TSG 100; CGTase–treated (1.0 × 10^−4^ U/g substrate).

The textural properties of RCF and CGTase–treated TSGs were evaluated by measuring hardness and cohesiveness (Fig. 4). Hardness decreased with increasing enzyme levels, with TSG 100 showing the lowest value (*P* < 0.05). TSG 40 and TSG 60 exhibited intermediate hardness values that did not differ significantly from each other, while TSG 80 showed a further reduction. In contrast, cohesiveness remained relatively constant across all samples, with no significant differences observed among treatments. All samples belonged to the same statistical group for cohesiveness.

### 3.5 Microstructural observation of gels by SEM

**Fig. 5:**
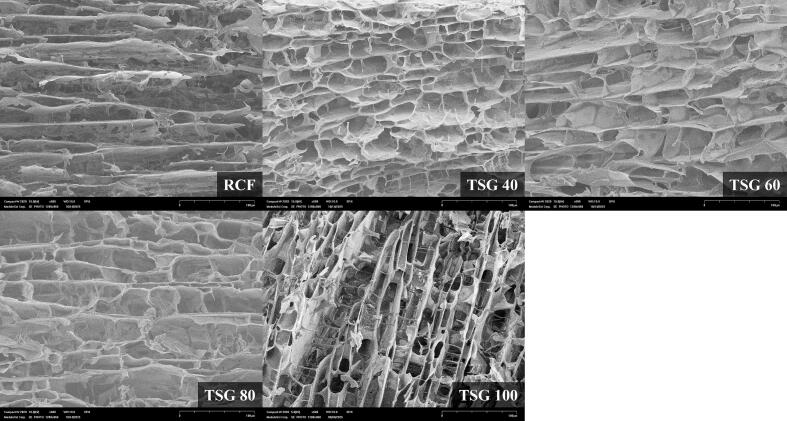
SEM micrographs of pore microstructure in freeze–dried gel cross–sections. Samples were observed at 500× magnification under accelerating voltages of 5 and 10 kV. RCF shows a dense and compact structure, while CGTase–treated samples exhibit progressively developed porous networks depending on enzyme level.

SEM analysis showed distinct differences in pore microstructure among RCF and CGTase–treated TSGs (Fig. 5). Untreated RCF exhibited a compact, layered morphology with no apparent pore formation, representing a dense gel structure. In contrast, TSG 40 and TSG 60 showed the development of relatively uniform and well–defined pores, suggesting that low to moderate levels of CGTase treatment loosened the gel matrix and initiated pore formation. TSG 80 displayed a more heterogeneous pore network characterized by wider variations in pore size, reflecting increased chain rearrangement and structural expansion at higher enzyme levels. TSG 100 demonstrated extensive pore formation along with partially collapsed wall structures and noticeably reduced wall thickness. Overall, pore morphology shifted from a dense, nonporous structure to more open and irregular networks with increasing CGTase levels.

### 3.6 Freeze–thaw stability

**Fig. 6:**
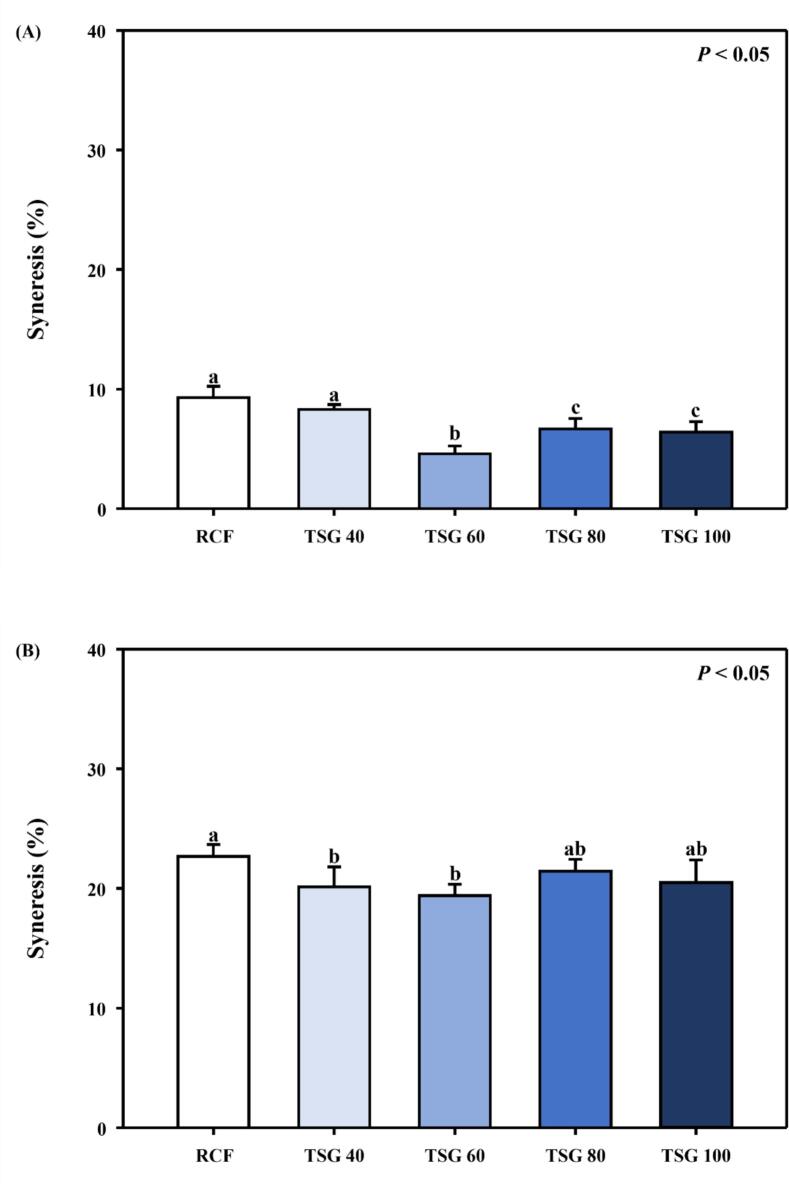

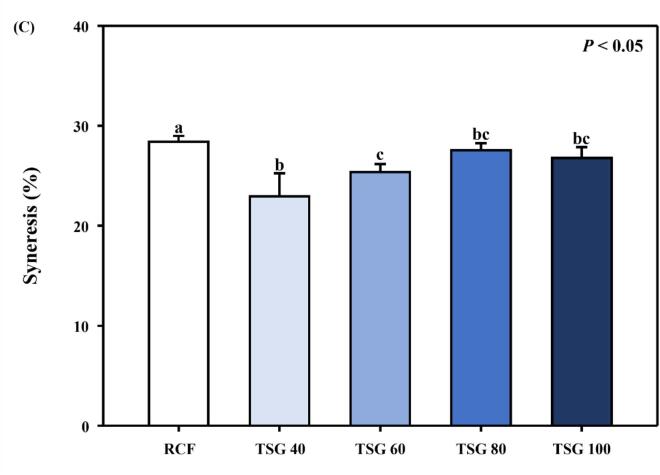
Syneresis of RCF and CGTase–treated TSGs during three freeze–thaw cycles. Syneresis values measured after the first (A), second (B), and third (C) freeze–thaw cycles. All measurements were conducted in triplicate under identical conditions.

Freeze–thaw stability was evaluated by measuring syneresis, expressed as the percentage of water released during each cycle. In the first cycle, syneresis values were 9.35% for RCF, 8.35% for TSG 40, 4.65% for TSG 60, 6.73% for TSG 80, and 6.47% for TSG 100, showing lower water separation in all enzyme–treated samples compared with RCF (Fig. 6A). In the second cycle, syneresis increased across all groups but remained lower in the enzyme–treated samples (20.18–21.50%) than in RCF (22.74%), as shown in Fig. 6B. By the third cycle, syneresis further increased, reaching 28.47% in RCF, whereas the CGTase–treated groups exhibited lower values of 23.00% for TSG 40, 25.42% for TSG 60, 27.61% for TSG 80, and 26.83% for TSG 100 (Fig. 6C). Overall, enzyme–treated samples showed lower syneresis than RCF across all cycles, with differences depending on enzyme level and cycle number.

## 4 Discussion

CGTase treatment induced structural modification of rice cake flour, resulting in altered rheological, textural, and freeze–thaw properties of the resulting gels. These effects are interpreted in relation to CGTase-mediated chain rearrangement and changes in starch molecular organization.

Temperature cycling revealed that CGTase–induced modification altered both the thermal resilience and recovery behavior of RCF gels (Fig. 1). Native RCF partially re–established its elastic network upon cooling, as evidenced by G′ recovery exceeding the initial modulus. In contrast, enzyme–treated samples exhibited greater decreases in G′ during heating and enzyme dose–dependent differences in recovery during cooling, indicating altered temperature–dependent rheological behavior. These effects likely arise from CGTase–catalyzed disproportionation, which alters starch chain organization within the gel network. HPAEC analysis showed an increased proportion of medium–length branches (DP 16–30) in enzyme–treated samples compared with untreated RCF (Fig. 3), indicating a shift in chain–length distribution. Such structural changes influence the balance between ordered and amorphous regions within the gel network, contributing to the decrease in G′ during heating and its recovery during cooling (Xiao et al., 2025; Zhang et al., 2020). This redistribution of starch chains may increase network flexibility while preserving sufficient intermolecular interactions, thereby contributing to reversible viscoelastic behavior during repeated heating–cooling cycles. In addition, for RCF and TSG 80, the storage modulus after repeated temperature cycles exceeded the initial value, suggesting partial retrogradation during cycling (Fig. 1) (Rezler, 2021). Under the applied reaction conditions, CGTase primarily promoted chain rearrangement reactions such as disproportionation, as supported by the observed changes in chain length distribution and molecular weight, while cyclodextrin formation was minimal and did not significantly contribute to the observed gel properties (Fig. S1). The cyclic variation of tan δ further supported reversible transitions between elastic and viscous behavior during repeated heating–cooling cycles (Fig. 2).

The molecular weight of the enzyme–treated samples, as assessed by HPSEC, was markedly lower than that of untreated RCF (Table 1). This reduction is consistent with the reactions catalyzed by CGTase, including cyclization, coupling, disproportionation, and hydrolysis, which promote transglycosylation–driven rearrangement of starch chains accompanied by partial cleavage of α–1,4 glycosidic linkages (Leemhuis et al., 2010). Disproportionation redistributes branch–chain lengths, whereas hydrolysis shortens chains, both contributing to the decrease in Mw at the macromolecular scale (Ji et al., 2020).

The relationship between DP distribution and Mw is inherently complex and cannot be interpreted linearly, as starch molecular architecture involves heterogeneous chain distributions and branching patterns (Bertoft, 2017). This is supported by HPAEC results showing an increase in medium–length branches (DP 16 to 30) and a decrease in short branches (DP 6 to 15) in enzyme–treated samples compared with untreated RCF (Fig. 3). These changes in branch–chain distribution influence intermolecular interactions, as medium–length chains are more effective in forming junction zones, contributing to the development of a more stable gel network. This molecular restructuring is consistent with the textural changes observed in TPA (Fig. 4), where modified chain architecture enhances network connectivity and mechanical integrity. These results indicate that the observed effects are primarily associated with structural rearrangement of starch chains rather than changes in amylose and amylopectin composition. In addition to starch structure, minor components such as proteins and lipids in RCF may influence enzymatic modification and gel formation by affecting enzyme accessibility or local structural rearrangement (Zhang et al., 2020). However, because all samples were derived from the same source and processed under identical conditions, the observed differences are mainly attributed to CGTase–mediated modification, although the contribution of these minor components cannot be completely excluded.

The observed changes in Mw are closely associated with the rheological and textural properties of TSGs. In starch–based gel systems, Mw governs chain entanglement and network formation, thereby influencing viscoelastic behavior and mechanical strength (Miao & BeMiller, 2023). An increase in Mw enhances intermolecular interactions and contributes to the formation of a more cohesive gel network, improving elasticity and firmness. However, gel properties are not determined solely by absolute Mw but are also strongly influenced by chain length distribution and molecular architecture. In this study, CGTase–mediated disproportionation redistributed glucan chains, generating a balanced distribution of shorter and longer chains that supports efficient network formation and viscoelastic stability. At higher enzyme concentrations, the apparent increase in Mw is likely associated with transglycosylation–driven chain elongation (Fig. 3). Although this can improve network connectivity, excessive chain length reduces chain mobility and limits network rearrangement by restricting segmental motion, thereby constraining further improvements in rheological behavior (Kobayashi et al., 1999). These findings demonstrate that Mw and chain length distribution must be considered together to understand the structure–property relationships of TSGs.

CGTase treatment induced a clear modification of the textural properties of TSGs, as evidenced by the progressive decrease in hardness from 2.53 N in RCF to 1.14 N in TSG 100 (Fig. 4A). This trend is consistent with the reduction in network strength that may arise from CGTase–mediated chain rearrangement and partial chain scission, including hydrolytic cleavage (Zheng et al., 2024). Such softening aligns with the observed changes in branch–chain distribution, where a decrease in short chains (DP 6–15) and an increase in intermediate chains (DP 16–30) would be expected to weaken junction zones and reduce gel rigidity. The gradual decline in hardness across enzyme levels further supports dose–dependent weakening of the gel matrix. In contrast, cohesiveness remained statistically unchanged across all samples, with no significant differences among treatments (Fig. 4B) (Nishinari et al., 2013). The maintenance of cohesiveness despite substantial reductions in hardness suggests that the fundamental connectivity of the gel network was preserved. The increased proportion of intermediate and long chains in CGTase–treated samples likely provides sufficient intermolecular interactions to maintain structural continuity even as overall firmness decreases. Taken together, these results indicate that CGTase treatment enables modulation of RCF gel texture by reducing hardness while retaining cohesiveness, resulting in gels that are softer while maintaining network integrity.

SEM micrographs provide direct insight into the structural consequences of CGTase treatment and integrate the physicochemical and functional changes observed in TSGs. Untreated RCF exhibited a dense, layered microstructure without discernible pores, consistent with its high hardness, high syneresis, and limited temperature–dependent rheological behavior. This compact morphology reflects a tightly packed starch network formed by intact long chains and short branching segments that maintain strong junction zones. In contrast, CGTase–treated samples showed progressively more open and porous structures with increasing enzyme levels, indicating substantial molecular reorganization. The formation of uniform pores in TSG 40 and TSG 60 corresponds to the reduction in Mw and the decrease in short branches (DP 6 to 15), which weaken intermolecular packing and reduce the density of crosslinking regions. These structural changes explain the moderate decrease in hardness and the improved freeze–thaw stability observed in these samples. Notably, TSG 60, which exhibited the lowest syneresis in the first cycle, displayed a pore structure that was open but not excessively disrupted, suggesting an optimal balance between network loosening and structural integrity. At higher enzyme levels, TSG 80 and TSG 100 showed more heterogeneous pore networks with thinner pore walls and partially collapsed structures. These features are consistent with the increased proportion of intermediate and long chains (DP ≥ 16), as well as the partial recovery of Mw at higher enzyme concentrations. The extensive pore development and wall thinning provide a structural basis for the further decrease in hardness. Despite these pronounced structural changes, cohesiveness remained unchanged across all treatments, indicating that fundamental network connectivity was preserved. This suggests that chain entanglement and residual branching interactions were sufficient to maintain overall gel continuity.

The freeze–thaw results also reflect these structural distinctions (Tang et al., 2018). While all CGTase–treated groups showed lower syneresis than RCF across cycles, the extent of improvement varied with microstructural features revealed by SEM. Samples with moderately expanded and uniform pore structures (TSG 40 and 60) exhibited superior freeze–thaw stability, whereas those with overly enlarged or partially collapsed pore walls (TSG 80 and 100) showed less pronounced improvements. These results indicate that not only pore formation but also pore uniformity and wall integrity play critical roles in water retention during freeze–thaw cycling. Taken together, the SEM findings demonstrate that CGTase treatment transforms the dense, nonporous RCF network into a more open, compliant, yet cohesive structure. This structural transition explains the observed decrease in hardness, the maintenance of cohesiveness, the redistribution of branch–chain lengths, the reduction in Mw, and the improvement in freeze–thaw stability. The combined effects of molecular rearrangement and controlled pore formation contribute to the development of TSGs exhibiting temperature–dependent rheological behavior, as reflected by the reversible changes in G′ during heating–cooling cycles (Fig. 1). The cyclic variation of tan δ further supports the reversible transition between elastic and viscous behavior during temperature cycling (Fig. 2). In contrast, TSG 80 exhibited partial over–recovery of G′ during cooling, but its broader pore size distribution and thinner pore walls limited freeze–thaw stability compared with TSG 40 and 60. These results suggest that TSG 80 may be more suitable for applications requiring elastic recovery or shape retention, whereas systems requiring high freeze–thaw stability may benefit from further optimization of structural uniformity. Further investigation using techniques such as low–field NMR could provide additional insights into water distribution within the gel network and clarify the mechanisms underlying freeze–thaw stability. In addition, although this study focused on enzyme dosage under fixed reaction conditions, other parameters such as reaction time and temperature may also influence CGTase–mediated structural modification and gel formation. Therefore, systematic studies considering multiple reaction parameters are required to fully understand their combined effects.

## 5 Conclusion

This study demonstrated that CGTase-mediated modification effectively transformed upcycled rice cake flour into TSGs with tunable structural and functional properties. Enzymatic treatment redistributed starch branch-chain lengths and altered molecular weight, contributing to reversible viscoelastic behavior, reduced hardness, and improved freeze–thaw stability. In particular, TSG 60 exhibited improved freeze–thaw stability, suggesting a favorable balance between network flexibility and structural integrity. These findings provide insight into how CGTase-mediated starch restructuring influences gel functionality and highlight a practical strategy for valorizing surplus rice cake byproducts into functional starch-based materials. The developed TSGs may have potential applications in thermo-processed and frozen food systems requiring improved textural and freeze–thaw properties.

## CRediT authorship contribution statement

**Da-Hee Kim:** Writing – original draft, Visualization, Methodology, Investigation, Conceptualization. **Tae-Ho Choi:** Visualization. **Ji-Hoon Park:** Investigation. **Su-Hyeon Kim:** Visualization. **Seo-Jin Jeon:** Visualization. **Hyun-Mo Jeong:** Methodology, Formal analysis, Conceptualization. **Jae-Hoon Shim:** Supervision, Resources, Project administration, Funding acquisition.

## Declaration of competing interest

The authors declare that they have no known competing financial interests or personal relationships that could have appeared to influence the work reported in this paper.

## Data Availability

Data will be made available on request.

## References

[bb0005] Bertoft E. (2017). Understanding starch structure: Recent progress. Agronomy.

[bb0010] Eom H., Chang Y., Lee E.-s., Choi H.-D., Han J. (2018). Development of a starch/gum-based edible coating for rice cakes to retard retrogradation during storage. LWT- Food Science and Technology.

[bb0015] Fan R., Cheng Y., Wang R., Zhang T., Zhang H., Li J., Zheng A. (2022). Thermosensitive hydrogels and advances in their application in disease therapy. Polymers.

[bb0020] Gong Y., Xiao S., Yao Z., Deng H., Chen X., Yang T. (2024). Factors and modification techniques enhancing starch gel structure and their applications in foods: A review. Food Chemistry: X.

[bb0025] Han J.A., Kwon K.H. (2023). Purchase behavior according to the development of sustainable pistachio (*Pistacia vera* L.) rice cake: For Korean consumers. Sustainability.

[bb0030] Ji H., Bai Y., Li X., Zheng D., Shen Y., Jin Z. (2020). Structural and property characterization of corn starch modified by cyclodextrin glycosyltransferase and specific cyclodextrinase. Carbohydrate Polymers.

[bb0035] Jin F., Kim S.-H., Choi Y.-K., Yoo B.-K. (2024). A study on the impact of hallyu (Korean wave) on Korea’s consumer goods exports to China: Panel analysis using big data and provincial-level data. Sustainability.

[bb0040] Karim A.A., Bhat R. (2008). Gelatin alternatives for the food industry: Recent developments, challenges and prospects. Trends in Food Science and Technology.

[bb0045] Kim E.-A., Lee Y.-R., Lee E.-H., Jeong H.-M., Kang B.S., Kim B.-H., Shim J.-H. (2023). Development and applications of enzymatic modified starch with high water solubility providing a continuous supply of glucose. International Journal of Biological Macromolecules.

[bb0050] Klouda L., Mikos A.G. (2008). Thermoresponsive hydrogels in biomedical applications. European Journal of Pharmaceutics and Biopharmaceutics.

[bb0055] Kobayashi M., Tsuzuki W., Funane K., Kato Y. (1999). Dormant activity of cyclodextrin glucanotransferase on dextran afforded the molecular changes. Journal of Applied Glycoscience.

[bb0060] Lee H.-S., Jang M.-S. (2008). The development of the HACCP plan in Korean rice cake manufacturing facilities. Korean Journal of Food and Cookery Science.

[bb0065] Lee H.-W., Jeon H.-Y., Choi H.-J., Kim N.-R., Choung W.-J., Koo Y.-S., Shim J.-H. (2016). Characterization and application of BiLA, a psychrophilic α-amylase from *Bifidobacterium longum*. Journal of Agricultural and Food Chemistry.

[bb0070] Lee K., Kim Y., Park K., Lee H. (2006). Effects of α-glucanotransferase treatment on the thermo-reversibility and freeze-thaw stability of a rice starch gel. Carbohydrate Polymers.

[bb0075] Leemhuis H., Kelly R.M., Dijkhuizen L. (2010). Engineering of cyclodextrin glucanotransferases and the impact for biotechnological applications. Applied Microbiology and Biotechnology.

[bb0080] Lv T., Chen Y., Li N., Liao X., Heng Y., Guo Y., Hu K. (2025). A comprehensive review of thermosensitive hydrogels: Mechanism, optimization strategies, and applications. Gels.

[bb0085] Mateos-Aparicio I., Matias A., Galanakis C.M. (2019). The role of alternative and innovative food ingredients and products in consumers wellness.

[bb0090] Miao M., BeMiller J.N. (2023). Enzymatic approaches for structuring starch to improve functionality. Annual Review of Food Science and Technology.

[bb0095] Moon H.-J., Cha Y.-S. (2023). Sustainability of K-food: Focused on the change in the health values of K-food. Journal of Ethnic Foods.

[bb0100] Nishinari K., Kohyama K., Kumagai H., Funami T., Bourne M.C. (2013). Parameters of texture profile analysis. Food Science and Technology Research.

[bb0105] Pardhi D.S., Rabadiya K.J., Panchal R.R., Raval V.H., Joshi R.G., Rajput K.N. (2023). Cyclodextrin glucanotransferase: Fundamentals and biotechnological implications. Applied Microbiology and Biotechnology.

[bb0110] Peira G., Bollani L., Giachino C., Bonadonna A. (2018). The management of unsold food in outdoor market areas: Food operators’ behaviour and attitudes. Sustainability.

[bb0115] Rezler R. (2021). Rheological analysis of the structuralisation kinetics of starch gels. Molecules.

[bb0120] Rhowell J.N.T., A. P. B, Nese S. (2021). Enhancing the functional properties of rice starch through biopolymer blending for industrial applications: A review. International Journal of Biological Macromolecules.

[bb0125] Seo N.S., Roh S.A., Auh J.H., Park J.H., Kim Y.R., Park K.H. (2007). Structural characterization of rice starch in rice cake modified by thermus scotoductus 4-α-Glucanotransferase (TSαGTase). Journal of Food Science.

[bb0130] Tang X., Liu N., Huang W., Cheng X., Wang F., Zhang B., Li Z. (2018). Syneresis rate, water distribution, and microstructure of wheat starch gel during freeze-thaw process: Role of a high molecular weight dextran produced by *Weissella confusa* QS 813 from traditional sourdough. Cereal Chemistry.

[bb0135] Torres-León C., Ramírez-Guzman N., Londoño-Hernandez L., Martinez-Medina G.A., Díaz-Herrera R., Navarro-Macias V., Ascacio-Valdes J. (2018). Food waste and byproducts: An opportunity to minimize malnutrition and hunger in developing countries. Frontiers in Sustainable Food Systems.

[bb0140] Varghese S., Awana M., Mondal D., Rubiya M., Melethil K., Singh A., Thomas B. (2022). Handbook of biopolymers.

[bb0145] Wang B., Zheng H., Yang Y., Bian X., Ma C., Zhang Y., Sun S. (2024). Effect of different chain-length fatty acids on the retrogradation properties of rice starch. Food Chemistry.

[bb0150] Wibisono D.A.S., Saw C.-Y., Wu T.-Y., Chau C.-F. (2025). Advancing industrial food byproduct management: Strategies, technologies, and progress in waste reduction. Processes.

[bb0155] Xiao Y., Kong H., Jiang Z., Li C., Ban X., Gu Z., Li Z. (2025). Thermo-reversible gel synthesized by 4-α-glucanotransferase with sol-gel transition tuned by subtle amylose manipulation. Food Hydrocolloids.

[bb0160] Zhang Z., Li E., Fan X., Yang C., Ma H., Gilbert R.G. (2020). The effects of the chain-length distributions of starch molecules on rheological and thermal properties of wheat flour paste. Food Hydrocolloids.

[bb0165] Zhao D., Zhang X., Zhang Y., Xu E., Yan S., Xu H., Li M. (2024). Recent advances in the fabrication, characterization and application of starch-based materials for active food packaging: Hydrogels and aerogels. Sustainable Food Technology.

[bb0170] Zheng Q., Li X., McClements D.J., Jin Z., Qiu C. (2024). Effects of cyclodextrin glycosyltransferase on physicochemical, digestion, and gel properties of corn and potato starches. Food Hydrocolloids.

