# Supplementary material for: Development of sustainable gel systems with thermo–sensitive properties by enzymatic modification of upcycled rice cake flour
Source: Food Chem X. 2026 Jun 12;37:104095. doi: 10.1016/j.fochx.2026.104095 (PMC13311792; doi:10.1016/j.fochx.2026.104095)
Supplement: Supplementary file 1 — Supplementary material [file mmc1.docx]

**Supplementary Data**

**
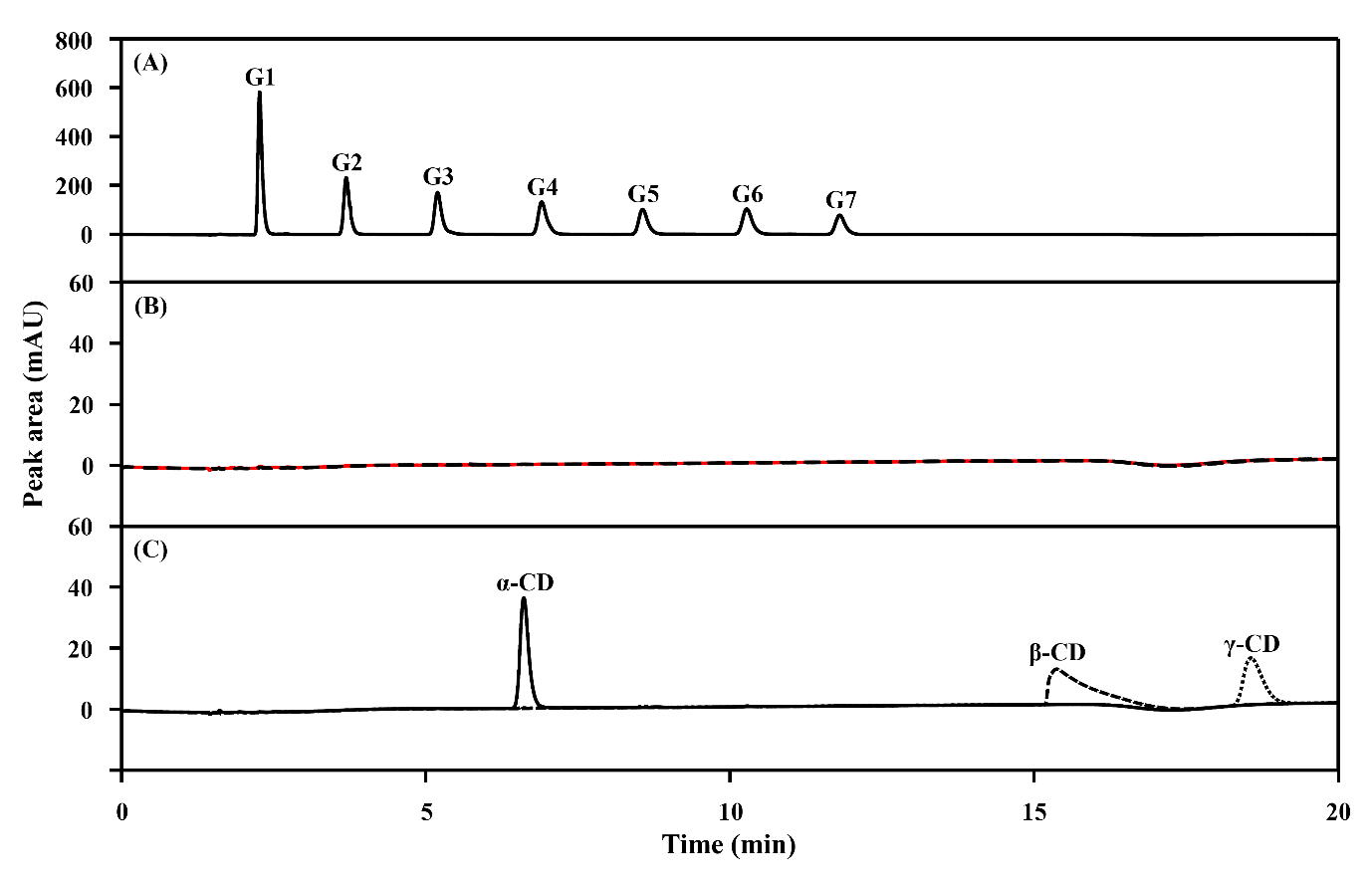
**

**Figure S1. HPAEC analysis for the detection of cyclodextrin formation in CGTase-treated samples.** HPAEC chromatograms showing (A) maltooligosaccharide standards (G1–G7), (B) untreated rice cake flour (RCF, red) and CGTase-treated samples (TSGs, black), and (C) cyclodextrin standards (α-CD, β-CD, and γ-CD). The chromatograms of RCF and TSGs did not exhibit detectable peaks corresponding to cyclodextrins, indicating negligible cyclodextrin formation under the applied reaction conditions.

**Table S1. Apparent amylose and amylopectin contents of RCF and CGTase-treated thermo-sensitive gels.** Values are expressed as mean ± SD (n = 3). Different superscript letters within a column indicate significant differences (P < 0.05) based on Tukey’s test.

| **Samples** | **Amylose (A)**  **(mg/mL)** | **Amylopectin (B)**  **(mg/mL)** | **Total contents**  **(mg/mL)** | **Ratio (B/A)** |
| --- | --- | --- | --- | --- |
| RCF | 0.053 ± 0.001ᵉ | 0.142 ± 0.001ᵉ | 0.195 | 2.69 ± 0.01ᵉ |
| TSG 40 | 0.038 ± 0.001ᶜ | 0.108 ± 0.001ᶜ | 0.146 | 2.81 ± 0.01ᶜ |
| TSG 60 | 0.038 ± 0.001ᵈ | 0.105 ± 0.001ᵈ | 0.143 | 2.75 ± 0.01ᵈ |
| TSG 80 | 0.045 ± 0.001ᵇ | 0.127 ± 0.001ᵇ | 0.172 | 2.84 ± 0.01ᵇ |
| TSG 100 | 0.049 ± 0.001ᵃ | 0.144 ± 0.001ᵃ | 0.193 | 2.92 ± 0.01ᵃ |

The apparent contents of amylose and amylopectin may be influenced by the water-holding capacity of the gel system, affecting absolute values. However, the amylose:amylopectin ratio is less sensitive to such variation and better reflects relative compositional changes. All CGTase-treated samples showed an increased ratio compared to RCF. This trend may be attributed to disproportionation, in which amylose is transferred to amylopectin side chains, leading to a relative decrease in amylose. Similar behavior has been reported for 4-glucosyltransferase (Seo et al, 2007). Structural rearrangement may also contribute, although its effect is considered limited.

* Seo, N.-S., Roh, S.-A., Auh, J.-H., Park, J.-H., Kim, Y.-R., & Park, K.-H. (2007). Structural characterization of rice starch in rice cake modified by *Thermus scotoductus* 4-α-glucanotransferase. *Journal of Food Science*, 72(6), C331–C336.
